# Residential green space quantity and quality and symptoms of psychological distress: a 15-year longitudinal study of 3897 women in postpartum

**DOI:** 10.1186/s12888-018-1926-1

**Published:** 2018-10-26

**Authors:** Xiaoqi Feng, Thomas Astell-Burt

**Affiliations:** 1 Population Wellbeing and Environment Research Lab (PowerLab), School of Health and Society, Faculty of Social Sciences, University of Wollongong, Wollongong, NSW Australia; 2 Menzies Centre for Health Policy, University of Sydney, Sydney, NSW Australia; 3 Illawarra Health and Medical Research Institute (IHMRI), University of Wollongong and Illawarra and Shoalhaven Local Health District, Wollongong, NSW Australia; 4 School of Public Health, Peking Union Medical College and the Chinese Academy of Medical Sciences, Beijing, China

**Keywords:** Mental health, Green space quality, Green space quantity, Women, Postpartum

## Abstract

**Background:**

Experiments and large-scale epidemiological studies indicate the importance of green space for mental health. However, little research has been conducted to elucidate whether these mental health benefits are more dependent upon the quantity or quality of the green space.

**Methods:**

Symptoms of psychological distress were measured in 3897 women who did not change neighbourhood up to 15 years postpartum using the Kessler 6 psychological distress scale from 2004 onwards. The percentage land-use of the neighbourhood was used to ascertain a measure of green space quantity. A Likert scale was used to measure green space quality in response to the statement “*there are good parks, playgrounds and play spaces in this neighbourhood.”* Multilevel negative binomial growth curve regression models were used to examine the patterning of symptoms of psychological distress across the postpartum period in relation to green space quantity and quality, adjusting for person-level and geographical markers of confounding. The same variables were also fitted in multilevel logistic regressions to examine the odds of reporting serious mental illness (as defined by K6 scores ≥ 13 out of 24).

**Results:**

Symptoms of psychological distress were fewer among women who agreed (rate ratio (RR) 0.95, 95%CI 0.91 to 0.98) and strongly agreed (RR 0.89, 95%CI 0.85 to 0.93) local parks were good quality. The odds of reporting serious mental illness were also lower among women who agreed (odds ratio (OR) 0.88, 95%CI 0.77 to 1.00) and strongly agreed (OR 0.74, 95%CI 0.64 to 0.86) local parks were good quality. No association was found between green space quantity and symptoms of psychological distress or the odds of reporting serious mental illness.

**Conclusions:**

This study suggests it may be how mothers perceive green space nearby and what those spaces enable them to do, rather than simply how much there is overall, that is important for promoting mental health in the postpartum period. In conclusion, community consultation is likely to be a crucial part of strategies that maximise the health benefits of urban greening for everyone.

## Background

Urban greening and restoration of green space is widely believed to promote mental health through a range of pathways. This is supported by evidence from experiments and large-scale observational studies [1–4] which suggest contact with green spaces (e.g. parks) provides effortless restoration and opportunities for stress reduction. This includes a range of studies suggesting views of nature promote memory recall [5], relaxed wakefulness [6], altered cerebral blood flow and brain activation patterns consistent with relaxation [7, 8]. Studies have suggested that physical (and probably also social) recreation in environments containing green space also reduces the risk of minor psychiatric morbidity [9, 10] and facilitates an enhanced sense of self and connectedness with nature [11]. This in turn stimulates forward-looking, pro-social thinking and reductions in future discounting associated with depression and negative health behaviours [12]. Other pathways may include natural soundscapes that enhance stress reduction and cognitive restoration [13], phytochemicals that might have benefits for immune and central nervous systems [14], higher levels of negative ions [15] that may have value for the treatment of depression [16] and increased exposure to lactic acid bacteria and microbial genera which are ubiquitous in the natural environment and could influence depression, fatigue, and cognition [17].

Reviewing many of the epidemiological studies conducted so far on adults in Australia [9, 18, 19], New Zealand [20], the UK [21–24], the US [25, 26], the Netherlands [27–29] and Denmark [30] reveals that the majority of evidence is based upon cross-sectional data and supports interventions that focus on increasing the quantity of residential green space. This conclusion is also supported by recent systematic reviews of research on adults and children [31, 32]. This is important to help set guidelines on how much green space is needed to promote mental health, given that opportunities for coming into contact with nature worldwide are becoming rarer as parks within cities and around the urban fringe are redeveloped into new housing, commercial premises and other forms of built environment. Within this context, however, even maintaining a certain quantity of green space may be very challenging and might ultimately take a lower priority in comparison to other needs. Evidence is required on not just how much green space is needed, but also what green space is fit for purpose.

‘Fit for purpose’ is a concept that pushes beyond quantity to notions of quality, which given its policy relevance is a surprisingly far less researched issue in epidemiological studies of green space and health. In fact, only three studies have examined the potential mental health benefit of quality green space in adult samples. One study in Perth (Australia) found lower odds of psychological distress among participants living near moderate to high quality green spaces, but no association with green space quantity [19]. A study of four cities in the Netherlands found evidence of mental health benefits of both green space quantity and quality, but with the strongest evidence for high quality greenery [29]. A third study in Santiago (Chile) reported better mental health among people living in areas where there were more green spaces viewed as appropriately maintained [33]. Each of these studies was reliant upon cross-sectional data and could not examine how green space quantity and quality might matter as people age, which is important as many studies have already shown that mental health varies across the lifecourse (e.g. [21]).

Accordingly, our aim was to answer the question ‘is green space quantity or quality more important for mental health?’ using a longitudinal study. We focussed upon adult women who were followed-up biennially from 0-1y up to 15y postpartum. The purpose of focussing upon women postpartum was to recognise an under-researched yet very large population subgroup within which depression is common and a significant cause of morbidity and mortality [34, 35]. For example, recent work suggested that 14.5% of women experienced symptoms of depression at 4 years postpartum, higher than at any point in the first 12 months following childbirth [36]. It may also be that green space quality matters more than quantity during this sensitive period of the lifecourse, given the needs of the mother and that it remains common for them to be responsible for the majority of childrearing activities. For example, it is plausible that for mothers of infants a small but high quality green space nearby that affords opportunities for outdoor relaxation and socialising within a short walk of home has greater restorative potential than a large amount of green space viewed as poor quality. With evidence from prior studies suggesting that there can be a discrepancy between subjective and objective distance to the nearest green space (e.g. [37]), it may be that nearby green spaces that are perceived to be low in quality may offer very little restorative benefit. As such, this sample was considered to provide an important test of whether a focus on green space quantity is sufficient, or if green space quality also matters for protecting mothers’ mental health in the postpartum period.

## Methods

Data on mothers in the postpartum period was obtained from Australia’s definitive birth cohort, the Longitudinal Study of Australian Children (LSAC). Permission to obtain and analyse the LSAC was granted through a formal application to The Department of Social Services of the Australian Government (the data custodian). The LSAC began in 2004 and involved biennial follow-up of approximately 10,000 children and at least one parent each. The majority of the parents who participated were mothers [38]. Sampling for the LSAC originated with the Australian Government’s provider of universal healthcare listing all permanent residents and citizens (‘Medicare’) in a two-stage clustered design. Representativeness of the sample across urban, regional and rural communities was obtained via sampling of postcodes stratified by state and territory and by capital city statistical division compared with the rest of the state in 2004. Recruitment and consent for participation was obtained via letters posted to parents who were randomly selected from the sample postcodes. The rate of recruitment was 50.4%, with 37.5% opting out and 15.2% being uncontactable. Data collection via face-to-face interviews had response rates > 90% at baseline and approximately 80% thereafter. Further information on the LSAC sampling and variables is available in a published data user guide [39].

This study used an accelerated cohort design [40] that made use of data on all mothers irrespective of whether their child had been classified as part of the (a) ‘baby cohort’, which was aged 0-1y at baseline, or the (b) ‘kindergarten’ cohort, aged 4-5y at baseline. Mother and child were tracked biennially for up to 4 more waves after baseline. We restricted the sample to only those mothers who did not change neighbourhood during this period, leaving 4259 (42.2%) participants in the sample from both cohorts. Further omission of participants who were not mothers and/or did not have a response on the K6 measure brought the final sample to 3897 women. By survey wave this was as follows: 2004 (*n* = 3526); 2006 (*n* = 3429); 2008 (*n* = 3191); 2010 (*n* = 3469); 2012 (*n* = 3425); and (2014 (*n* = 3367).

### Mental health

Symptoms of psychological distress were measured using the Kessler 6 Psychological Distress Scale (K6) in each wave [41, 42]. The K6 is a short form screening instrument for non-specific psychological distress based upon responses to six questions as follows: *“During the last 30 days, how often did:* (1) *you feel nervous?;* (2) *you feel hopeless?;* (3) *you feel restless or fidgety?;* (4) *you feel so depressed that nothing could cheer you up?;* (5) *you feel that everything was an effort?;* and (6) *you feel worthless?”* Responses were rated as 0 = “none of the time”, 1 = “a little of the time”, 2 = “some of the time”, 3 = “most of the time” and 4 = “all of the time”. In the current study, the sum of all six responses was used as the outcome variable, in order to examine incremental changes that may occur across the postpartum period. The score was scaled with a base value of 0, ranging up to 24. Assessment of the odds of serious mental illness was defined using scores of 13 or greater out of 24 [43].

### Green space measures

Different sources of data were used to measure green space quantity and quality. The quantity of green space was measured according to the percentage of the land-use of the ‘Statistical Area 2’ of residence covered in ‘parkland’ according to the Australian Bureau of Statistics (ABS). This land-use category did not include agricultural land. Data on green space was extracted from the ABS’s 2006 meshblock classification and aggregated to the Statistical Area 2 unit, which was designed by the ABS with populations of 10,000 on average (ranging from 3000 to 25,000) and to represent local communities and functional areas that contain commercial and transport hubs in areas outside of cities [44]. Mother’s self-reports were used to identify the availability of good quality green space nearby. This involved reclassifying Likert scale responses to the statement: *“there are good parks, playgrounds and play spaces in this neighbourhood”* into ‘did not agree’, ‘agreed’ and ‘strongly agreed’. In specifying whether a mother felt good parks and similar spaces were available nearby, this focussed on quality and avoided simply detecting whether any green space was proximal. This is an important caveat as some previous work with a different dataset in Australia has reported lower odds of psychological distress among people living nearby higher quality green space, but no association between psychological distress and the quantity of green space per se [19].

### Confounding

Socioeconomic and geographical factors play an important role in determining mental health [45] and also likely influence access to more and better quality green space [46–48]. In this study, mother’s economic status and her highest educational qualification were taken into account. Economic status includes whether a mother was employed, unemployed, not working and not looking for a job (‘economically inactive’) or other categories, such as a student. Aboriginal and Torres Strait Islander status was also taken into account as women of these ethnic groups are known to disproportionately greater levels of socioeconomic disadvantage [49]. The geographic context where each woman lived was expressed in two different ways. First, the Accessibility/Remoteness Index of Australia was used to differentiate between mothers living in major cities, inner regional, outer regional, remote and very remote communities [50]. Second, strata of area-level socioeconomic circumstances were incorporated using the Socio Economic Index For Areas (SEIFA) relative index of social disadvantage [51], which is a composite measure of census-based indicators including local income, educational attainment and employment rates.

### Statistical analysis

The percentage of the sample at high risk of psychological distress were described across the postpartum period and in relation to other sample characteristics. Multivariate analysis was then conducted using multilevel negative binomial regression models in MLwIN V3.00 [52]. Each model explicitly took into account variation in symptoms of psychological distress across three levels: (1) person-year observations (i.e. time); (2) person; (3) neighbourhood (Statistical Area 2). Negative binomial regressions were used to assess symptoms of psychological distress, to account for over-dispersed integer counts. Parameter estimates were expressed as rate ratios (RR). Separate models were used to examine association between symptoms of psychological distress with measures of green space quantity and quality, before and after adjusting for confounding. Mean-centred linear and square terms of age were fitted to account for potential non-linearities. A final set of models were fitted with a two-way interaction term between the number of years postpartum (also mean-centred linear and square terms to account for potential non-linearities) and each of the green space measures, to investigate whether mean associations with symptoms of psychological distress were consistent across the postpartum period. All of these analyses were repeated using multilevel logistic regression of the odds of serious mental illness, with parameters expressed as odds ratios (OR).

## Results

The overall sample of 3897 included 20,407 observations nested within 859 areas and a prevalence of serious mental illness (K6 ≥ 13) at 12.4%. Table 1 reports how this prevalence was distributed by each of the sample characteristics using chi-square values. The prevalence of serious mental illness appeared to vary by only about 1.4% between mothers living in areas with ≤ 5% green space compared with those who had ≥ 41%. In contrast, compared to those who felt that there were no good quality parks nearby 16.0% K6 ≥ 13, those who agreed (12.8%) or strongly agreed (9.7%) that there were good parks nearby had a lower prevalence of serious mental illness. Some variation in the prevalence of serious mental illness was observed with respect to years since childbirth. Lower prevalence was noted with age, higher educational qualifications, among those who were employed, and living in more affluent areas. Higher prevalence was observed among mothers who identified as Aboriginal or Torres Strait Islander, economically inactive or unemployed. There appeared to be little variation in the prevalence of serious mental illness with respect to geographic remoteness.

**Table 1:** Sample counts and percentage reporting serious mental illness, as defined by scores ≥ 13 out of 24 on the Kessler 6 Psychological Distress Scale, across sample characteristics

	N	% K6 ≥ 13	Chi2 (*p*-value)
Total	20,407	12.4	
Green space quantity			
< 5%	4204	11.7	
6–10%	3292	11.9	
11–20%	5671	13.7	
21–40%	4987	12.9	
> 41%	2253	10.3	22.2 (< 0.001)
Parks good quality			
Do not agree	3726	16.0	
Agree	9626	12.8	
Strongly Agree	6548	9.7	
No response	507	13.4	89.2 (< 0.001)
Years since childbirth			
0-1y	1658	14.2	
2-3y	1623	8.8	
4-5y	3362	15.4	
6-7y	3440	11.0	
8-9y	3312	11.7	
10-11y	3436	12.1	
12-13y	1810	12.6	
14-15y	1766	12.9	60.4 (< 0.001)
Age group			
17 to 29	1004	16.4	
30 to 34	3198	13.8	
35 to 39	6007	12.3	
40 to 44	6084	12.0	
45 to 63	4114	11.2	26.9 (< 0.001)
Indigenous status			
No	20,108	12.3	
Yes	290	18.6	
No response	9	33.3	14.1 (0.001)
Highest educational qualification			
Postgraduate	3357	10.5	
Undergraduate	12,172	12.7	
Year 11 to 13	3172	11.4	
≤ Year 10	1693	16.1	
Other	13	23.1	38.3 (< 0.001)
Economic status			
employed	14,994	10.4	
economically inactive	4979	17.5	
unemployed	419	23.6	
no response	15	13.3	224.2 (< 0.001)
Area disadvantage			
affluent	6786	10.3	
average	6745	12.4	
deprived	6876	14.5	54.3 (< 0.001)
Geographic remoteness			
major cities	13,165	12.9	
inner regional	4331	11.1	
outer regional or remote	2911	12.2	9.9 (0.007)

**Fig. 1:**
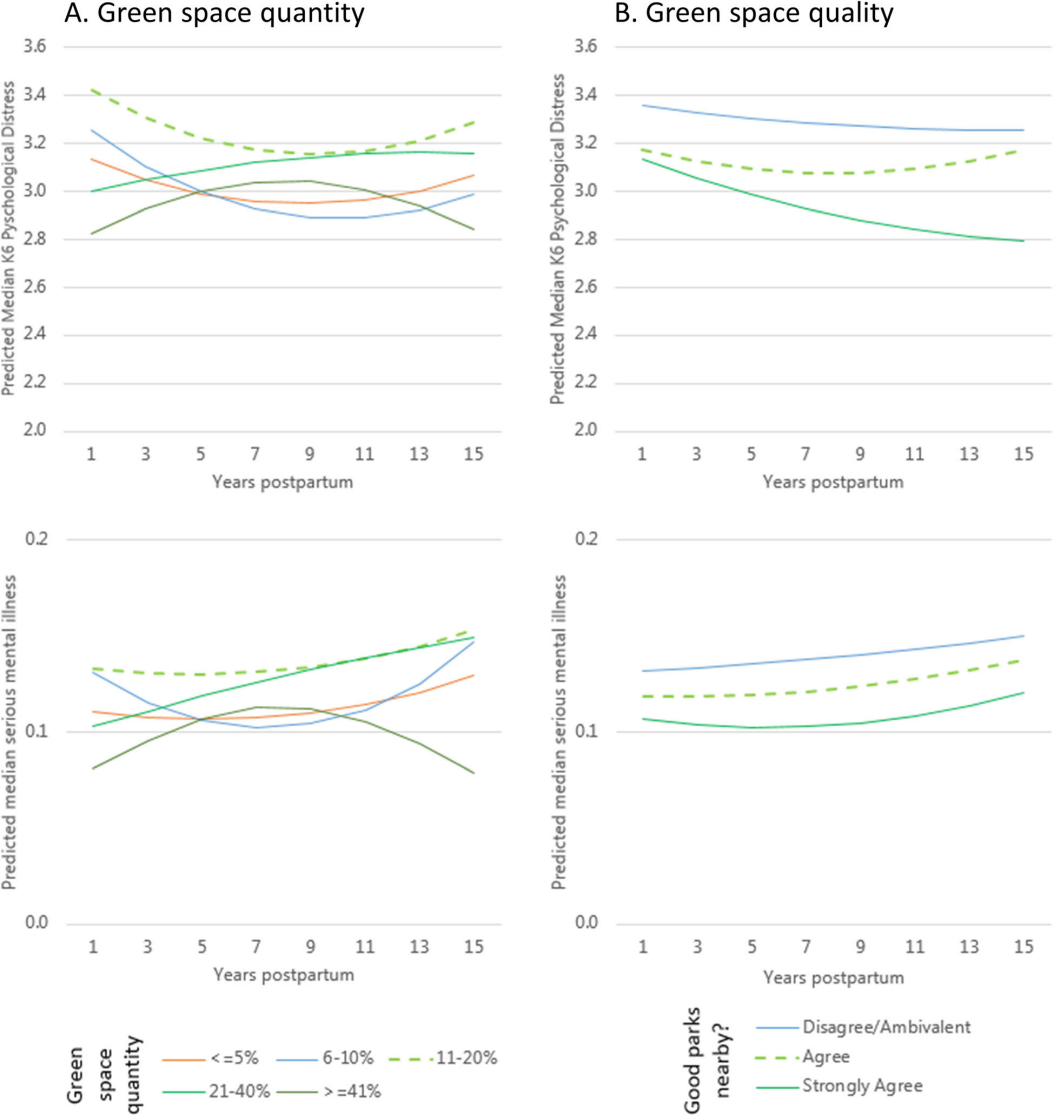
Median Kessler 6 Psychological Distress Scale and predicted median serious mental illness among women in the postpartum period by the number of years since childbirth, in relation to green space (**a**) quantity and (**b**) quality, predicted from multilevel negative binomial and logistic regression models adjusted for confounding

In adjusted multilevel negative binomial regressions there was no convincing evidence of an average effect of green space quantity on symptoms of psychological distress, nor of an interaction between green space quantity and the number of years since childbirth (Table 2, Models 1 and 2). Substituting the measure of green space quantity for that of green space quality yielded different results. Mothers who agreed (OR 0.95, 95%CI 0.91 to 0.98) or strongly agreed (OR 0.89, 95%CI 0.85 to 0.93) that local parks were of good quality had fewer symptoms of psychological distress than their counterparts who disagreed (Table 2, Model 3). There was no evidence that this association varied with respect to the number of years since childbirth (Table 2, Model 4). These patterns with respect to green space quantity and quality were also observed when switching from symptoms of psychological distress to the odds of serious mental illness as the study outcome (Table 3, Models 5 to 8). The non-significant trajectories in both outcomes for all of the final models featuring two-way interaction terms between the number of years since childbirth and each of the green space measures are illustrated in Fig. 1.

**Table 2:** Multilevel negative binomial regression models of symptoms of psychological distress in association with green space quantity and quality among women in the postpartum period

	Green space quantity		Green space quality	
	Model 1	Model 2	Model 3	Model 4
Fixed part	Rate Ratio (95% Confidence Interval)			
Age (mean centred)	0.99 (0.98, 0.99)	0.99 (0.98, 0.99)	0.99 (0.98, 0.99)	0.99 (0.98, 0.99)
Age square (mean centred)	1.00 (1.00, 1.00)	1.00 (1.00, 1.00)	1.00 (1.00, 1.00)	1.00 (1.00, 1.00)
Indigenous status (No)				
Yes	1.30 (1.06, 1.59)	1.30 (1.06, 1.59)	1.30 (1.06, 1.59)	1.30 (1.06, 1.59)
Highest qualification (ref: Postgraduate)				
Undergraduate	1.01 (0.95, 1.07)	1.01 (0.95, 1.07)	1.01 (0.95, 1.07)	1.01 (0.95, 1.07)
Year 11 to 13	0.94 (0.87, 1.01)	0.93 (0.86, 1.01)	0.93 (0.86, 1.01)	0.93 (0.86, 1.01)
≤ Year 10	1.02 (0.93, 1.12)	1.02 (0.93, 1.12)	1.02 (0.93, 1.11)	1.02 (0.93, 1.12)
Other	1.34 (0.63, 2.84)	1.34 (0.63, 2.84)	1.39 (0.65, 2.95)	1.39 (0.66, 2.96)
Economic status (ref: Employed)				
Economically inactive	1.11 (1.07, 1.14)	1.11 (1.07, 1.15)	1.11 (1.07, 1.14)	1.11 (1.07, 1.14)
Unemployed	1.10 (1.02, 1.19)	1.10 (1.02, 1.19)	1.10 (1.01, 1.19)	1.10 (1.01, 1.19)
Area disadvantage (ref: affluent)				
Average	1.04 (1.00, 1.09)	1.04 (1.00, 1.09)	1.03 (0.99, 1.08)	1.03 (0.99, 1.08)
Disadvantaged	1.08 (1.03, 1.13)	1.08 (1.03, 1.13)	1.06 (1.01, 1.11)	1.06 (1.01, 1.11)
Remoteness (ref: Major Cities)				
Inner regional	0.91 (0.85, 0.98)	0.91 (0.85, 0.98)	0.89 (0.83, 0.96)	0.89 (0.83, 0.96)
Outer regional or remote	0.94 (0.87, 1.03)	0.94 (0.87, 1.03)	0.93 (0.85, 1.01)	0.93 (0.85, 1.01)
Years since childbirth (mean centred)	1.00 (0.99, 1.01)	1.00 (0.98, 1.01)	0.99 (0.98, 1.01)	1.00 (0.98, 1.01)
Years since childbirth^2^ (mean centred)	1.00 (1.00, 1.00)	1.00 (1.00, 1.01)	1.00 (1.00, 1.00)	1.00 (1.00, 1.01)
Green space quantity (ref: ≤ 5%)				
6–10%	0.99 (0.90, 1.09)	0.98 (0.89, 1.09)		
11–20%	1.07 (0.99, 1.16)	1.07 (0.98, 1.17)		
21–40%	1.04 (0.96, 1.13)	1.06 (0.97, 1.16)		
≥ 41%	0.99 (0.89, 1.09)	1.03 (0.92, 1.15)		
Parks good quality (ref: Do not agree)				
Agree			0.95 (0.91, 0.98)	0.94 (0.90, 0.98)
Strongly Agree			0.89 (0.85, 0.93)	0.88 (0.84, 0.93)
Years since childbirth × Green space quantity				
Years × 6–10%		0.99 (0.97, 1.01)		
Years × 11–20%		1.00 (0.98, 1.01)		
Years × 21–40%		1.01 (0.99, 1.03)		
Years × ≥ 41%		1.00 (0.98, 1.02)		
Years since childbirth^2^ × Green space quantity				
Years^2^ × 6–10%		1.00 (0.99, 1.01)		
Years^2^ × 11–20%		1.00 (0.99, 1.01)		
Years^2^ × 21–40%		1.00 (0.99, 1.00)		
Years^2^ × ≥ 41%		0.99 (0.98, 1.00)		
Years since childbirth ×Parks good quality				
Years × Agree				1.00 (0.99, 1.02)
Years × Strongly agree				0.99 (0.97, 1.01)
Years since childbirth^2^ × Parks good quality				
Years^2^ × Agree				1.00 (0.99, 1.01)
Years^2^ × Strongly agree				1.00 (0.99, 1.01)
Random part				
Level 3: Statistical Area 2	0.004 (0.006)	0.004 (0.006)	0.004 (0.006)	0.004 (0.006)
Level 2: Person	0.558 (0.016)	0.559 (0.016)	0.557 (0.016)	0.557 (0.016)

Level 1: Observation (*N* = 20,407) | Level 2: Person (*N* = 3897) | Level 3: Statistical Area 3 (*N* = 859)

**Table 3:** Multilevel logistic regression models of the odds of serious mental illness in association with green space quantity and quality among women in the postpartum period

	Green space quantity		Green space quality	
	Model 5	Model 6	Model 7	Model 8
Fixed part	Odds Ratio (95% Confidence Interval)			
Age (mean centred)	0.98 (0.97, 0.99)	0.98 (0.97, 0.99)	0.98 (0.97, 0.99)	0.98 (0.97, 0.99)
Age square (mean centred)	1.00 (1.00, 1.00)	1.00 (1.00, 1.00)	1.00 (1.00, 1.00)	1.00 (1.00, 1.00)
Indigenous status (No)				
Yes	1.55 (0.96, 2.50)	1.55 (0.96, 2.51)	1.58 (0.98, 2.55)	1.58 (0.98, 2.56)
Highest qualification (ref: Postgraduate)				
Undergraduate	1.11 (0.93, 1.32)	1.11 (0.93, 1.32)	1.10 (0.92, 1.31)	1.10 (0.92, 1.30)
Year 11 to 13	0.92 (0.73, 1.15)	0.91 (0.73, 1.14)	0.91 (0.73, 1.14)	0.91 (0.73, 1.14)
≤ Year 10	1.26 (0.97, 1.63)	1.26 (0.97, 1.63)	1.23 (0.95, 1.59)	1.23 (0.95, 1.59)
Other	1.92 (0.28, 13.23)	1.91 (0.28, 13.06)	2.10 (0.31, 14.14)	2.10 (0.31, 14.10)
Economic status (ref: Employed)				
Economically inactive	1.47 (1.31, 1.65)	1.47 (1.31, 1.66)	1.47 (1.31, 1.66)	1.47 (1.31, 1.65)
Unemployed	1.68 (1.27, 2.22)	1.68 (1.27, 2.23)	1.68 (1.27, 2.22)	1.67 (1.26, 2.22)
Area disadvantage (ref: affluent)				
Average	1.23 (1.07, 1.42)	1.24 (1.07, 1.42)	1.19 (1.04, 1.37)	1.19 (1.03, 1.37)
Disadvantaged	1.47 (1.25, 1.72)	1.47 (1.25, 1.72)	1.37 (1.17, 1.60)	1.36 (1.16, 1.60)
Remoteness (ref: Major Cities)				
Inner regional	0.75 (0.63, 0.89)	0.75 (0.63, 0.89)	0.71 (0.59, 0.84)	0.71 (0.59, 0.84)
Outer regional or remote	0.79 (0.64, 0.97)	0.79 (0.64, 0.97)	0.76 (0.62, 0.93)	0.76 (0.62, 0.93)
Years since childbirth (mean centred)	1.03 (0.99, 1.07)	1.03 (0.97, 1.09)	1.02 (0.99, 1.06)	1.02 (0.97, 1.08)
Years since childbirth^2^ (mean centred)	1.00 (0.99, 1.01)	1.01 (0.99, 1.03)	1.00 (0.99, 1.01)	1.00 (0.98, 1.02)
Green space quantity (ref: ≤ 5%)				
6–10%	1.01 (0.81, 1.27)	0.94 (0.72, 1.22)		
11–20%	1.24 (1.02, 1.51)	1.25 (1.00, 1.57)		
21–40%	1.16 (0.95, 1.42)	1.22 (0.97, 1.55)		
≥ 41%	0.89 (0.69, 1.14)	1.04 (0.78, 1.39)		
Parks good quality (ref: Do not agree)				
Agree			0.88 (0.77, 1.00)	0.86 (0.73, 1.02)
Strongly Agree			0.74 (0.64, 0.86)	0.71 (0.59, 0.87)
Years since childbirth×Green space quantity				
Years × 6–10%		0.99 (0.92, 1.07)		
Years × 11–20%		1.00 (0.93, 1.07)		
Years × 21–40%		1.03 (0.97, 1.11)		
Years x ≥ 41%		0.97 (0.88, 1.06)		
Years since childbirth^2^ × Green space quantity				
Years^2^ × 6–10%		1.02 (0.99, 1.05)		
Years^2^ × 11–20%		1.00 (0.97, 1.03)		
Years^2^ × 21–40%		0.99 (0.96, 1.02)		
Years^2^ × ≥ 41%		0.96 (0.92, 1.00)		
Years since childbirth × Parks good quality				
Years × Agree				1.00 (0.95, 1.06)
Years × Strongly agree				1.00 (0.94, 1.07)
Years since childbirth^2^ × Parks good quality				
Years^2^ × Agree				1.00 (0.98, 1.03)
Years^2^ × Strongly agree				1.01 (0.98, 1.04)
Random part				
Level 3: Statistical Area 2	0.005 (0.032)	0.004 (0.032)	0.009 (0.032)	0.009 (0.032)
Level 2: Person	2.195 (0.096)	2.199 (0.096)	2.184 (0.096)	2.181 (0.096)

Level 1: Observation (*N* = 20,407) | Level 2: Person (N = 3897) | Level 3: Statistical Area 3 (*N* = 859)

## Discussion

The key finding from this longitudinal study is that green space quality, but not quantity, is associated with fewer symptoms of psychological distress and lower odds of serious mental illness among women up to 15 years postpartum. The second key finding was that the potential effect of green space quality and non-effect of green space quantity on mental health appeared to be relatively consistent across this period of time in the women’s lives. To our knowledge, this is only the fourth study to have examined associations between mental health and measures of green space quantity and quality. It is the first to do so longitudinally. It is also the first study to investigate for evidence of mental health benefits of green space among women in postpartum, a period that can pose significant psychological challenges. Most of the research on green space and health in or around this important lifecourse stage focusses upon birth outcomes such as birthweight [53]. Just two studies have been published on green space and symptoms of psychological distress in pregnant women, each focussing on quantity-based measures and finding mixed results [54, 55]. This study therefore highlights and begins to address an important gap in understandings of green space and maternal health.

The findings from this study align relatively closely with another set in a different area of Australia on a general population sample, which also found evidence of a mental health benefit from proximity to quality green space, but not the availability of green space per se [19]*.* The results of both Australian studies contrast with evidence from the Netherlands where both measures of quantity and quality seemed to matter [29]. A sample size of four studies prohibits any definitive conclusions, though it may be that similarities between the Australian studies and contrasts with the Dutch findings reflect the potential for international variation in the relationship between green space quantity, quality and mental health, as well as geographical differences in how people value and interact with green spaces of varying quality. Geographical differences may be indicative of a range of aspects including cultural, socioeconomic and historical factors, variations in urban form, and climatic and topographical influences. There is also potentially important variation in the availability of green spaces within and between countries that intersect with other components of the built and natural environment (e.g. green walls, linear parks along river corridors) that may play a role, but were not specifically measured in our study. Given these possibilities, generalisations with regards to green space quality and mental health, or other health outcomes, to other countries should be only with the utmost caution. To advance the field of enquiry, there is a need for research specifically designed to examine the issue of green space quality in different countries simultaneously, not only to describe likely variations with mental health and other relevant outcomes, but also to learn about success stories in urban greening and green space restoration strategies, in order to identify what elements could be transferable across national boundaries and to promote population health in other contexts.

The results of this study with respect to green space quantity were unexpected, given the multiple pathways by which green space quantity may influence mental health. Green space quality has been found to be more important than green space quantity in another study of mental health with different data in Australia [19]. However, other studies have found green space quantity to be relevant. For example, one study used the same green space land-use data (though not the same metric) to report lower odds of psychological distress with more green space in a sample of men and women aged 45 years and older [9]. Unlike the aforementioned study, however, the geographical unit of analysis in our sample was the Statistical Area 2, which may be too large to identify associations with symptoms of psychological distress. It is plausible that the size of and distance to the nearest green space is particularly important for mothers due to well-known high levels of dependency upon parents during childhood [56]. Our measure of green space quantity may be an insufficient marker in this respect. Although all mothers in the data remained in the same location throughout the course of the study period, it is possible that in some cases the green space land-use (measured in 2006) could have changed over that time.

It is important to acknowledge potential circularity in the association between symptoms of psychological distress and self-reported green space quality. It is also plausible that in some contexts there may be good quality playgrounds and play spaces that are not set within some form of green space. Other features of the local environment not examined in this study may also play a confounding or modifying role, such as differences in walkability and places other than green spaces that mothers of young children may walk to, and potentially come into contact with green space as part of their journeys. Lastly, potentially mediating pathways linking green space quality and mental health in the postpartum period warrant dedicated investigation using longitudinal data and causal mediation models [57].

## Conclusions

If the results are interpreted as reflecting potentially causal relationships, this longitudinal study has provided evidence that green space quality matters for protecting mental health among women in the postpartum period. By contrast, the amount of green space nearby was not associated with fewer symptoms of psychological distress. Although this study is not without limitations, it seems reasonably clear from the findings of this study and just a few others before it to have examined mental health in relation to measures of green space quality, that efforts to redesign and restore green spaces need to involve community consultation in order to optimise the health benefits. Further research on green space quality and mental health generally and among women in the postpartum period is warranted to better understand what aspects of green space quality matter when, where and for whom.
